# Supplementary material for: A Humanized Yeast Phenomic Model of Deoxycytidine Kinase to Predict Genetic Buffering of Nucleoside Analog Cytotoxicity
Source: Genes (Basel). 2019 Sep 30;10(10):770. doi: 10.3390/genes10100770 (PMC6826991; doi:10.3390/genes10100770)
Supplement: Supplementary file 1 [file genes-10-00770-s001.zip › Additional_Files/Additional_File7_GOTermHeatmaps.zip!/Additional_File7_GOTermHeatmaps/rRNA_binding.pdf]

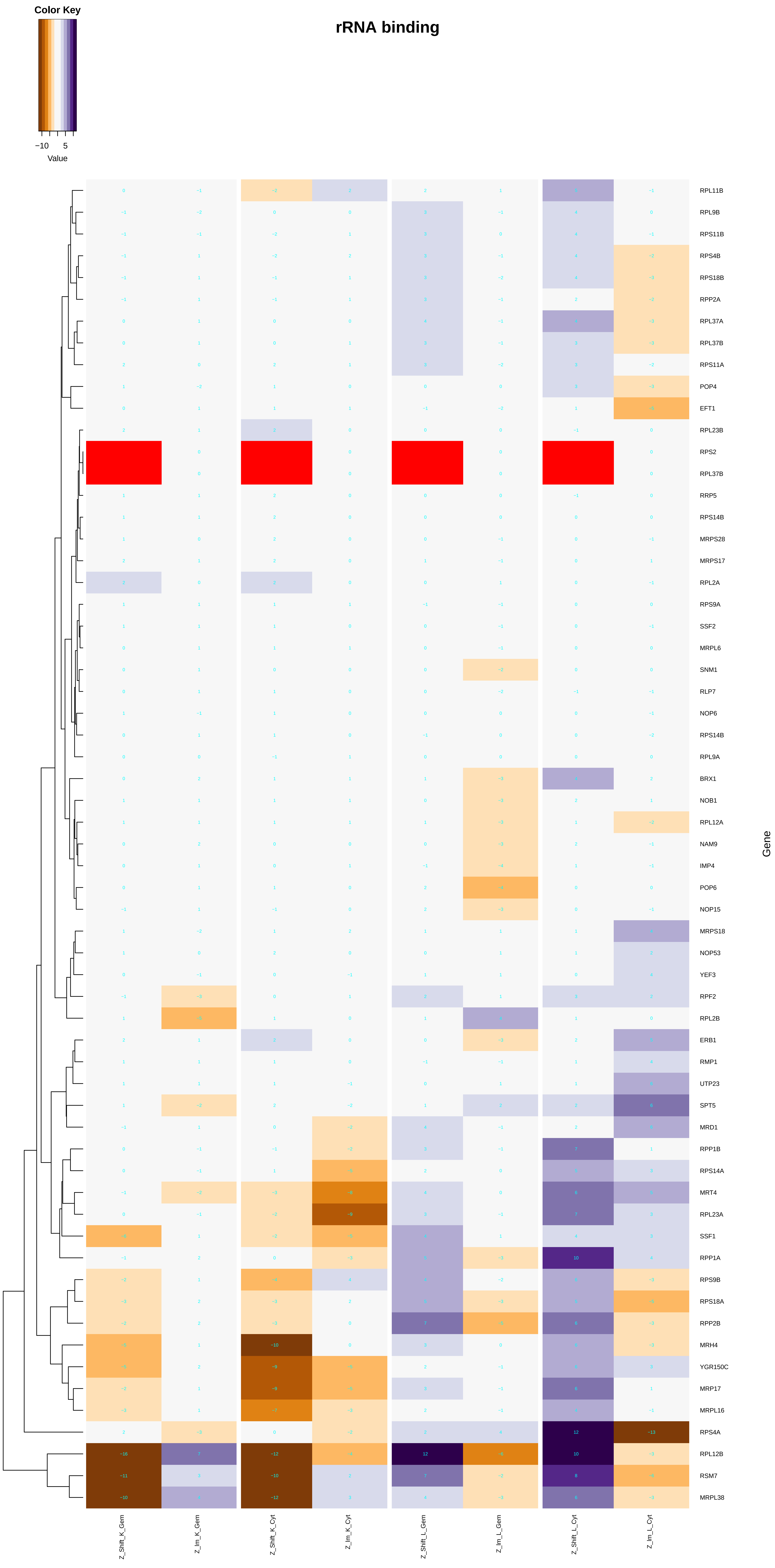
Color Key
−10
5
Value
rRNA binding
RPL11B
RPL9B
RPS11B
RPS4B
RPS18B
RPP2A
RPL37A
RPL37B
RPS11A
POP4
EFT1
RPL23B
RPS2
RPL37B
RRP5
RPS14B
MRPS28
MRPS17
RPL2A
RPS9A
SSF2
MRPL6
SNM1
RLP7
NOP6
RPS14B
RPL9A
BRX1
NOB1
RPL12A
NAM9
IMP4
POP6
NOP15
MRPS18
NOP53
YEF3
RPF2
RPL2B
ERB1
RMP1
UTP23
SPT5
MRD1
RPP1B
RPS14A
MRT4
RPL23A
SSF1
RPP1A
RPS9B
RPS18A
RPP2B
MRH4
YGR150C
MRP17
MRPL16
RPS4A
RPL12B
RSM7
MRPL38
Gene
Z_Shift_K_Gem
Z_Im_K_Gem
Z_Shift_K_Cyt
Z_Im_K_Cyt
Z_Shift_L_Gem
Z_Im_L_Gem
Z_Shift_L_Cyt
Z_Im_L_Cyt

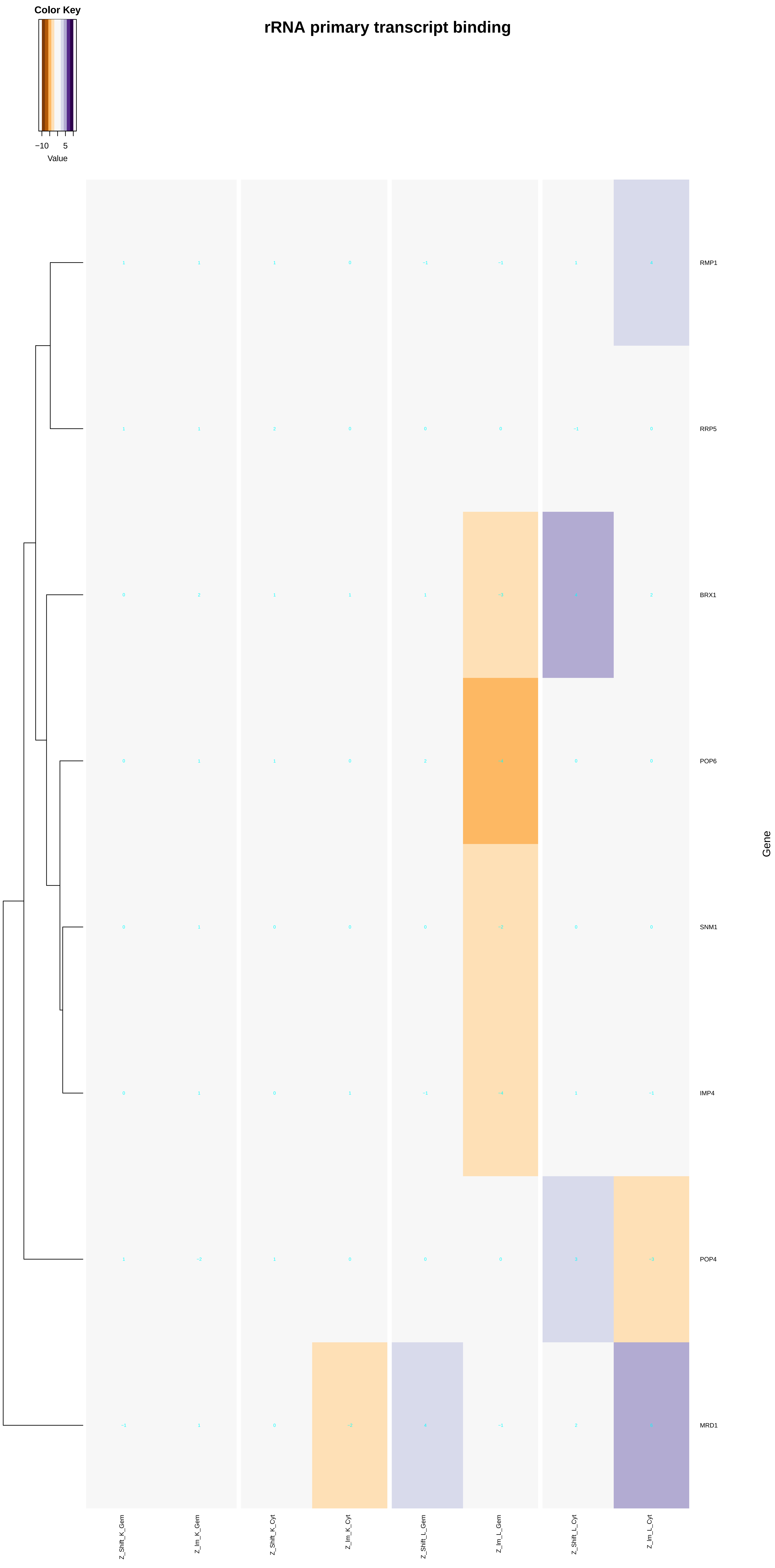
Color Key
−10
5
Value
rRNA primary transcript binding
RMP1
RRP5
BRX1
POP6
SNM1
IMP4
POP4
MRD1
Gene
Z_Shift_K_Gem
Z_Im_K_Gem
Z_Shift_K_Cyt
Z_Im_K_Cyt
Z_Shift_L_Gem
Z_Im_L_Gem
Z_Shift_L_Cyt
Z_Im_L_Cyt

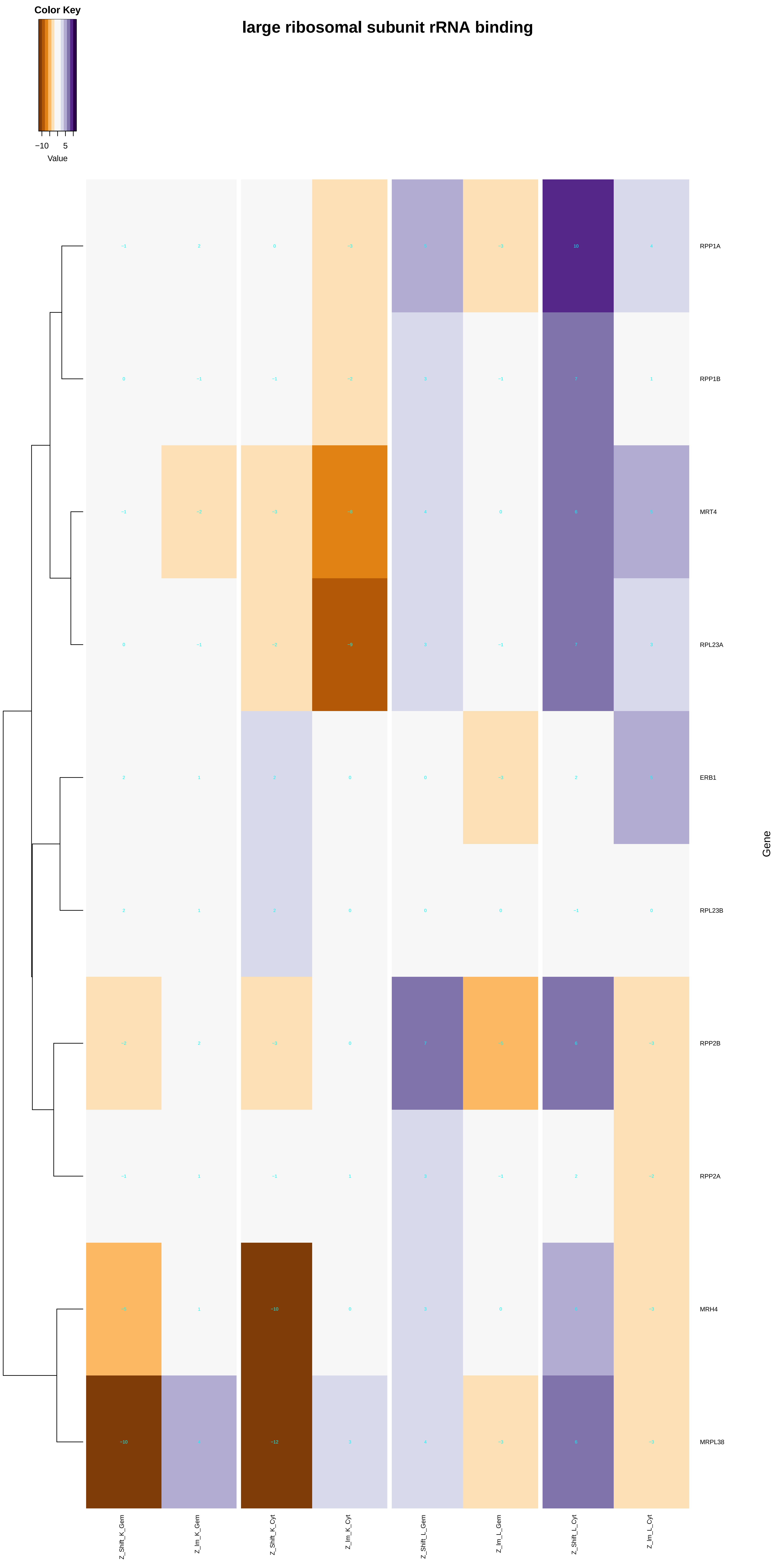
large ribosomal subunit rRNA binding
Color Key
−10
5
Value
RPP1A
RPP1B
MRT4
RPL23A
ERB1
RPL23B
RPP2B
RPP2A
MRH4
MRPL38
Gene
Z_Shift_K_Gem
Z_Im_K_Gem
Z_Shift_K_Cyt
Z_Im_K_Cyt
Z_Shift_L_Gem
Z_Im_L_Gem
Z_Shift_L_Cyt
Z_Im_L_Cyt

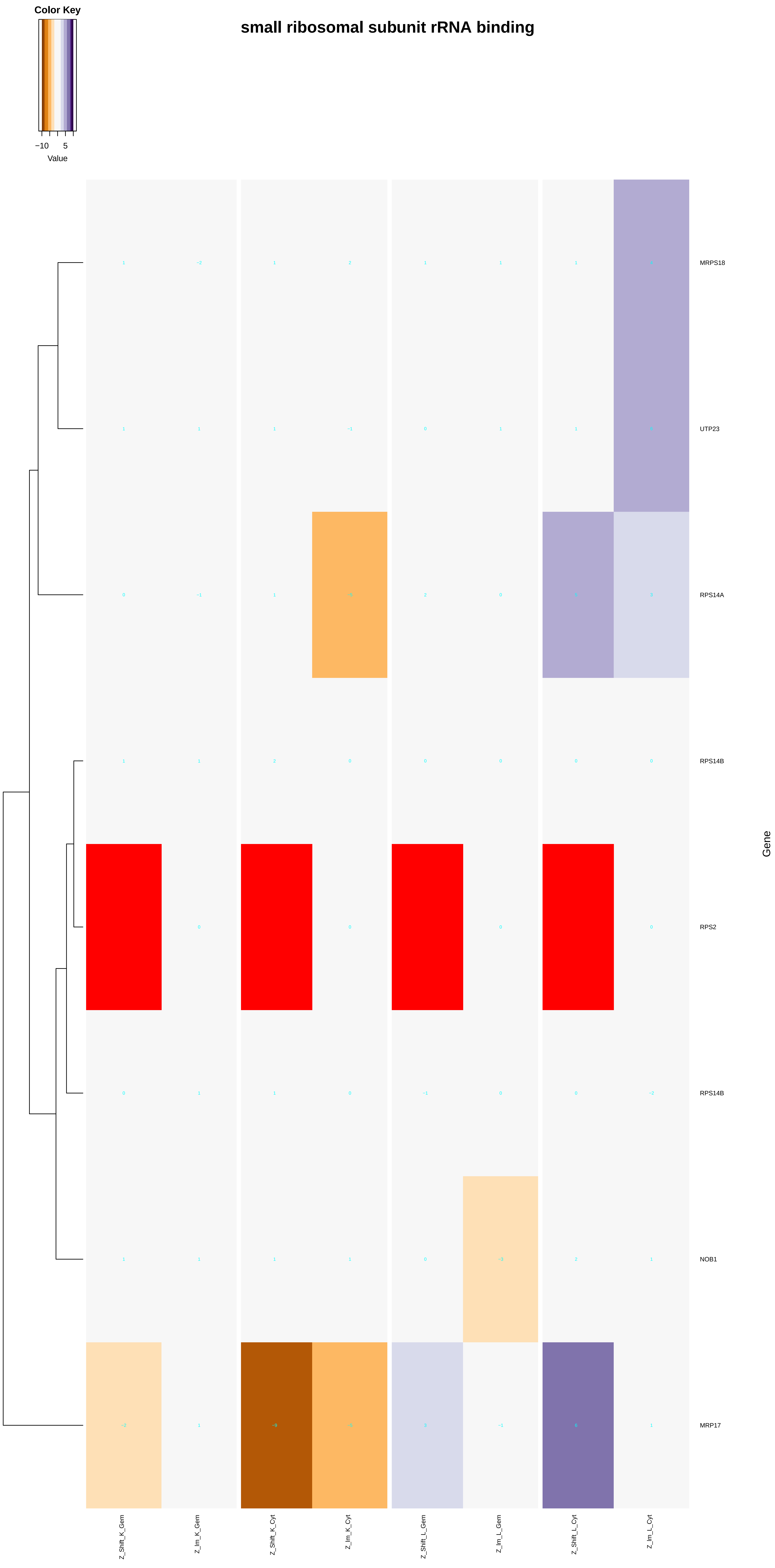
Color Key
−10
5
Value
small ribosomal subunit rRNA binding
MRPS18
UTP23
RPS14A
RPS14B
RPS2
RPS14B
NOB1
MRP17
Gene
Z_Shift_K_Gem
Z_Im_K_Gem
Z_Shift_K_Cyt
Z_Im_K_Cyt
Z_Shift_L_Gem
Z_Im_L_Gem
Z_Shift_L_Cyt
Z_Im_L_Cyt
